# *Piscirickettsia salmonis* pathogenicity: using the damage-response framework to look beyond smoke and mirrors

**DOI:** 10.1128/mbio.03821-24

**Published:** 2025-03-17

**Authors:** Felipe C. Cabello, Ana Millanao, Henry P. Godfrey

**Affiliations:** 1 Department of Pathology, Microbiology and Immunology, New York Medical College, Valhalla, New York, USA; 2 Department of Biological and Chemical Sciences, Faculty of Medicine and Science, Universidad San Sebastián, Valdivia, Chile; Albert Einstein College of Medicine, Bronx, New York, USA

**Keywords:** *Piscirickettsia salmonis*, fish pathogens, virulence, host-pathogen interactions, opportunistic infections, antibiotic resistance, environmental microbiology

## Abstract

*Piscirickettsia salmonis* is a globally distributed aquatic bacterium and a component of the normal salmon microbiome. It has significant biological and economic impact on Chilean salmon aquaculture due to the highly fatal disease, piscirickettsiosis. Unsuccessful attempts to prevent and treat this disease have resulted in heavy use of antimicrobials with adverse effects on the aquatic environment and piscine and human health. Evidence suggests *P. salmonis* could be a bacterium with relative pathogenic potential on farmed salmonids and other fishes that triggers piscirickettsiosis under particular conditions in the salmon and its environment. Application of a damage-response framework analysis could define the steps from asymptomatic *P. salmonis* infection to symptomatic disease, help tailor improved approaches to disease prevention and management, and, in turn, help avoid heavy use of antimicrobials which have global effects on animal health, human health, and environmental biodiversity (the One Health concept).

## OPINION/HYPOTHESIS

*Piscirickettsia salmonis* significantly impacts salmon aquaculture, mainly in Chile and particularly in Northern Patagonia, causing high mortality and $700 million in annual losses due to its association with salmonid piscirickettsiosis (1–5). This has resulted in *P. salmonis* being seen as a highly virulent and aggressive obligate salmon pathogen acquired via the intestine, gills, and damaged skin (1–5). It infects Atlantic salmon (*Salmo salar*), coho salmon (*Oncorhynchus kisutch*), and rainbow trout (*Oncorhynchus mykiss*), the significant species under aquaculture in Chile, as well as other salmonids such as Chinook salmon (*Oncorhynchus tshawytscha*). A comprehensive review did not support *P. salmonis* as an obligate pathogen but instead suggested it might be a bacterium with disease-causing potential that was resident in the environment and/or the normal salmon microbiome but did not consistently produce disease (6). The fact that *P. salmonis* is present in Norway, the world’s largest salmon producer, and in other salmon-producing countries without generating extended epizootics, significant economic losses, and heavy antimicrobial use is consistent with this suggestion (7–9).

Specific practices and environmental factors in Chilean salmon farming could contribute to triggering *P. salmonis* multiplication and outbreaks of piscirickettsiosis there (3, 6, 10). These include immunosuppression resulting from stress related to the passage of salmon from freshwater to seawater and high concentrations of caged fish under culture. They also include continuous use of large amounts of slowly degrading antimicrobials with resulting dysbiosis in both salmon and the environment. Between 2000 and 2020, Chilean salmon aquaculture used approximately 997 tons of quinolones, 3,051 tons of tetracyclines, and 4,375 tons of florfenicol to prevent and treat *P. salmonis* infections and piscirickettsiosis (6, 10, 11). In 2020 alone, 493 tons of florfenicol were imported in Chile for veterinary use (364 tons for aquaculture) (11), while not quite 0.1 tons (4,930 times less) of chloramphenicol, a related phenicol, were imported for human use (10). In 2023, there was a decrease in aquaculture use, with around 339 tons of antimicrobials, mainly florfenicol, used in production of around 1.1 million tons of salmonids (10–12). In Norway, in contrast, only 0.548 tons of these drugs were used in production of around 1.2 million tons of salmon, about 685 times less per million tons of fish produced (7, 8, 11, 12). Roughly 80% of antimicrobials used in aquaculture pass into the aquatic environment in a geographically limited area. There they accumulate, remain active for long periods, and select and help disseminate resistant pathogenic and non-pathogenic bacteria and their genes.

Sustainable growth and development of Chilean salmon aquaculture depends on clarifying the role of *P. salmonis* in piscirickettsiosis and finding approaches to prevent and treat it without using large amounts of antimicrobials (3, 4, 6, 10–12). Determining how this use affects global animal and human health and environmental biodiversity (the One Health model) is equally vital since such heavy use favors capturing novel resistance genes from the environment by piscine and human microbiomes (6, 10). In this opinion/hypothesis, we describe some gaps that need to be filled to approach this problem under the holistic damage-response framework (DRF) (Table 1 ) (13, 14).

**TABLE 1:** DRF and MISTEACHING as a guide for future studies to prevent *P. salmonis* infections and piscirickettsiosis in salmon^*a*^

Factor	Background	Possible approaches
Microbiome	A dysbiotic microbiome is present in salmon with piscirickettsiosis (5, 6).	1. Determine experimentally factors (stress, antimicrobial use, nutrition, immune response, co-infections) triggering dysbiosis in the salmonid microbiome undergoing piscirickettsiosis and favor *P. salmonis* multiplication and piscirickettsiosis. 2. Employ appropriate microbiological and genomics methods to confirm/rule out that *P. salmonis* is a standard component of the salmonid microbiome. 3. Identify microbiome components and networks that limit/favor the multiplication of *P. salmonis* in the salmon intestine, gills, and skin and mucous membranes. 4. Characterize and compare salmon microbiomes in Chile (high frequency of piscirickettsiosis) and Norway (low frequency of piscirickettsiosis) (5, 15).
Immunity	The immune response to *P. salmonis* is incompletely characterized. Preliminary evidence suggests granuloma formation with necrosis that damages multiple organs (3, 16).	1. Improve characterization of salmon immune responses to *P. salmonis* infection. This should encompass innate, humoral, cell-mediated responses, tolerance to microbiome components, and tissue damage. 2. Identify *P. salmonis* antigens mediating these responses which can block infection and multiplication of *P. salmonis in vitro* and in the host for use as potential vaccines.
Sex	Aquaculture in Chile uses mostly female salmon for biological and economic reasons, but female salmon are highly susceptible to *P. salmonis* infection (3, 4, 17, 18). Discovering whether males are more resistant to disease and the development of piscirickettsiosis could help analyze individual genetic determinants of this resistance.	Determine susceptibility of male salmon to *P. salmonis* infection and development of piscirickettsiosis, and identify genetic determinants underlying this resistance to allow their manipulation to resist *P. salmonis* infection and the development of piscirickettsiosis.
Temperature	*P. salmonis* infections and piscirickettsiosis are somewhat less common in waters of Southern Patagonia (average temperature around 9°) than in Northern Patagonia (average temperature around 14°) (19).	Determine the role of water temperature in facilitating *P. salmonis* infection and triggering piscirickettsiosis. This may help predict the development of piscirickettsiosis in a particular area.
Environment	Salmon are cultured at high concentrations in individual cages clustered in limited areas (6, 10). These practices could facilitate piscirickettsiosis development and transmission of *P. salmonis*.	1. Obtain data to confirm these practices can facilitate the development of piscirickettsiosis and transmitting *P. salmonis*. 2. Examine factors (eutrophication, anoxia, algal blooms, water temperature, marine dysbiosis produced by antimicrobials, metals) that may generate stress in salmon and facilitate the development of piscirickettsiosis. 3. Compare salmon culture conditions in Chile and Norway, industries with drastic differences in the frequency of piscirickettsiosis and the use of antimicrobials (6–8, 11, 12)
Age	The frequency of piscirickettsiosis increases in farmed salmon as they move from freshwater to the ocean and with time spent in the sea (3, 4, 6). Age may also influence the salmon’s immune response (16), and growth in a limited cage space in the ocean increases stress and mechanical skin lesions that favor *P. salmonis* entry and piscirickettsiosis development.	1. Determine the influence of age on the salmon immune responses. 2. Ascertain the metabolic adaptations that permit salmon survival when they move from freshwater to seawater and favor the development of piscirickettsiosis . 3. Establish the influence of age on the composition of the salmon microbiome, including the presence of *P. salmonis,* because age-related variations may predict the development of piscirickettsiosis.
Chance	Mutation and horizontal gene transfer may influence the selection of antimicrobial resistance in *P. salmonis* and modulate the expression of putative virulence genes. Chance environmental variations can increase stress, favoring piscirickettsiosis development and the diffusion of *P. salmonis* among different cages and groups of cages under these conditions.	1. Determine the frequency of genetic variation in *P. salmonis* and in salmon. 2. Use models to predict environmental variations (currents, winds, sea warming, and algal blooms) and use their results to avoid stress that favors piscirickettsiosis and the environmental spread of *P. salmonis*.
History	Various historical factors might influence the evolution of piscirickettsiosis in the 3-year cycle of salmon aquaculture. Characterizing their effect on this evolution may help prevent piscirickettsiosis.	Characterize effects on the evolution of piscirickettsiosis in smolts grown in different freshwater sources, exposure to eutrophication and algal blooms, treatment with antimicrobials able to produce dysbiosis, exposure to pathogens and co-infections that provide heterologous immunity and immunosuppression.
Inoculum	Farmed salmon with piscirickettsiosis may shed *P. salmonis* in sufficiently high concentrations to overcome the defenses of even healthy salmon.	1. Verify infection rates in salmon with various concentrations of *P. salmonis*. 2. Determine infection rates in uninfected salmon after co-culture with infected salmon. 3. Monitor temporal concentrations of *P. salmonis* in seawater.
Nutrition	The effects of reducing animal proteins in the diets of farmed salmon on the development of piscirickettsiosis are unknown. Passage of excessive feed into the aquatic environment leads to its eutrophication. Whether the ability of *P. salmonis* to regulate the metabolism of iron and other metals is associated with nutritional immunity in salmon is unknown.	1. Monitor whether decreasing animal proteins in the diet of farmed salmon favors the development of piscirickettsiosis through immunological, metabolic, and microbiome alterations in them. 2. Investigate whether eutrophication of the aquatic environment favors development of piscirickettsiosis mediated by immunological and metabolic alterations in salmon. 3. Determine if the ability of *P. salmonis* to regulate the metabolism of iron and other metals is associated with salmon nutritional immunity. 4. Compare diet composition in Chile and Norway.
Genetics	No unique Chilean pathotypes have been discovered to explain the high frequency of piscirickettsiosis in Chilean Patagonia (3, 4, 10).	1. Genomic characterization of additional *P. salmonis* isolates from Chile and other countries may confirm the lack of Chilean pathotypes or find them and characterize new mechanisms of virulence and antimicrobial resistance. 2. Comparative genetic characterization of farmed and wild salmon to identify determinants affecting immunologic and metabolic functions contributing to resistance and susceptibility to piscirickettsiosis and to vaccine failure.

^*a*^ MISTEACHING, microbiome, immunity, sex, temperature, environment, age, chance, history, inoculum, nutrition, and genetics.

It is still not known if *P. salmonis* is an externally acquired organism causing disease only under salmon-specific biological conditions, a standard component of the salmon microbiome that contributes to disease when the host-microbiome equilibrium breaks down, or both (Table 1) (4–6, 10). Knowledge is also lacking regarding the genetic, metabolic, and virulence differences between *P. salmonis* strains from Chile and those from Norway and Ireland, where, as mentioned above, *P. salmonis* is present but piscirickettsiosis is uncommon (4–6, 9, 10). The role of conditions that favor uncontrolled *P. salmonis* proliferation and piscirickettsiosis in salmon in Chile, including age, smolt quality, microbiome, sex, immunosuppression, and culture (high densities in pens, large numbers of pens in a limited geographical area, deficient nutrition, stress, temperature), needs better characterization (3–6, 10). Analysis of the management, veterinary medicine, and biosafety differences between Chilean and Norwegian aquaculture industries that could explain the striking differences in frequency and relevance of piscirickettsiosis and in antimicrobial use should be performed (6–10). The genetic and functional factors responsible for the failure of many different vaccine platforms to immunize salmon against *P. salmonis* and the nonperformance of tetracycline and florfenicol to prevent and treat piscirickettsiosis also need assessment (3–6).

### *P. salmonis* genomics and production of piscirickettsiosis

Initial and subsequent studies of *P. salmonis* have demonstrated worldwide distribution of its closely related LF, EM, and NC genospecies in the presence or absence of salmon farms, both in aquaculture areas where piscirickettsiosis is uncommon and in healthy salmon (3–6, 20). Genomic differences among isolates appeared irrelevant to host range, geographical distribution, and disease association. LF and EM were widely distributed in Chile; NC was present in four Canadian and Norwegian isolates. There was little gene recombination between genospecies and no specific Chilean pathotypes in any. Co-isolation of LF and EM genospecies from individual *S. salar* and *O. kisutch* in 3 different years in Chile suggested that *P. salmonis* might be a component of the normal salmon microbiome, consistent with its continued isolation from juvenile salmonids in freshwater (4, 21–23).

*P. salmonis* genomes contained 290 extrachromosomal genogroup-specific elements, few potential virulence-related factors, and mostly lacked antimicrobial resistance genes. This suggested minimal genetic exchange by horizontal gene transfer. Several LF genospecies harbored a previously described plasmid containing multiple antimicrobial resistance genes (4, 6, 24), while 67% of the 33 LF genomes of Chilean strains contained chromosomal mutations in the *gyrA* gene (4), most likely related to 10 years of heavy aquacultural quinolone use in this region (4, 6, 10). None of these findings could explain the unique severity of piscirickettsiosis in Chile. The wide distribution of *P. salmonis* in marine environments in areas where piscirickettsiosis is uncommon and the lack of any unique potential virulence properties in the genomes of genotypes present in Chile strengthen the hypothesis that *P. salmonis* is a potential disease-associated organism in the salmon microbiome and/or acquired externally that can trigger disease in the presence of underlying conditions present in Chile in the salmon and the environment rather than a strict pathogen (4, 6, 10, 13, 14).

### How pathogenic is *P. salmonis*?

The assumption that *P. salmonis* is an absolute pathogen rests on (i) its ability to express genes in its genome for virulence factors found in other bacteria, (ii) virulence experiments utilizing imperfect *in vitro* disease models, and (iii) production of piscirickettsiosis with appreciable mortality in fish infected with *P. salmonis*, mainly in Chile (3, 4, 6, 10). The presence of *Piscirickettsiaceae* and *P. salmonis* in healthy salmon and other fishes both in Chile and in other salmon aquaculture regions undermines its being an absolute pathogen (4–6, 10, 25, 26), but is consistent with a role as a potential one (4–6, 10, 13, 14).

The free-living residence of *P. salmonis* in saltwater and its culturability in cell-free media (3–6) undercut speculations of a preferred pathogenic intracellular location based on the 20% correspondence of genes in the *P. salmonis* genome to pseudogenes (4). Other evidence for absolute virulence factors in *P. salmonis* remains weak. Its Dot/Icm secretion system cluster has only 10 effector genes and displays many frameshift mutations and genetic rearrangements that may interfere with its expression, while the comparable system in *Legionella* has over 300 (4, 6, 27). This suggests this system is probably less relevant to virulence in *P. salmonis* than it is in *Legionella* (4, 27). Furthermore, the injection model used to assess the lack of infectivity of a *P. salmonis* Dot/Icm mutant may not replace the most appropriate current models, cohabitation, and immersion (3, 4, 6). Additional possible virulence factors (ClpB and BipA proteins; production of outer membrane vesicles, siderophores, and the ferric uptake regulator Fur; expression of putative toxin residues of unknown functions; formation of biofilms and small colonies) could easily be responsible for *P. salmonis* environmental survival, ability to colonize salmon, and residence in the microbiome of salmon and other fishes, with its being associated with disease only under particular conditions (3, 4, 6, 10, 13, 14, 25, 26).

Virulence is a complex emergent property whose determination cannot be based only on expression of one or multiple potential virulence factors while ignoring host and environmental factors (6, 10, 14, 20). Stanley Falkow, developer of molecular Koch’s postulates, recognized this: “The shared properties of commensals and pathogens of the same host species can be extraordinarily close and a source of consternation when trying to define a pathogen” (28).

### *P. salmonis*: a potential pathogen present in the normal salmon microbiome

*P. salmonis* is often present in healthy salmon and other fishes as a microbiome component associated with the disease only under particular host and environmental conditions (4–6, 10, 29, 30). Despite its presence in the mucosa and intestinal digests of both healthy salmon and salmon with piscirickettsiosis as detected by PCR of 16S RNA regions V4-V5, there were differences in the microbiomes of these two groups (5). This suggests that microbiome components limit or favor *P. salmonis* multiplication in salmon on a background of specific bacterial metabolic networks and that its presence and detection may be influenced by many factors (age, diet, antimicrobials), and that the salmon microbiome may also differ in different locations (5, 15, 31–35). While these studies confirmed the presence of dysbiosis in fish with piscirickettsiosis, it was impossible to conclude whether this dysbiosis caused or resulted from conditions permitting unrestricted proliferation of *P. salmonis* and the development of piscirickettsiosis (5, 6, 31–35). Moreover, microbiome studies in salmon may be limited by the scant biomass content of salmon gut (15, 32).

Some salmon with putative piscirickettsiosis skin lesions lacked intestinal *P. salmonis*, indicating it may in some cases be acquired from the environment via the skin (5, 6). While healthy salmon did not have *P. salmonis* in their skin, other potential piscine pathogens (*Aeromonas salmonicida*, *Tenacibaculum maritimum*, *Renibacterium salmoninarum*) were present in their intestines, and *Aliivibrio wodanis* and *Vibrio* spp. were detected in the gills and skin ulcerations of salmon with piscirickettsiosis (5, 35). Assuming sampling contamination can be ruled out, carrying bacteria in the piscine microbiome that can potentially cause disease may be a significant general and previously undescribed phenomenon only made evident by genomics and aquaculture (5, 35).

### *P. salmonis* virulence, piscirickettsiosis, and the DRF model

Proximity of salmon pens to one another facilitates *P. salmonis* transmission after its unrestricted multiplication and shedding in piscirickettsiosis. While this explains the geographical distribution of the disease (4, 6, 10), other conditions in the salmon and the environment that can trigger piscirickettsiosis remain uncharacterized (3–6, 10). This suggests that application of Casadevall and Pirofski’s DRF model (13, 14, 29, 30) to *P. salmonis* virulence could help define the steps from asymptomatic infection to symptomatic disease with the movement of salmon from freshwater to ocean culture and help create knowledge to tailor approaches to prevent and manage potential disease formation.

The DRF model considers pathogenicity an emergent and dynamic property resulting from interactions between would-be pathogen, host, and environment, with the levels of host damage arising from interactions between microorganisms and hosts. Such damage may be due to intrinsic microbial factors, host immune responses, or both. Casadevall and Pirofski categorized these interactions into six classes based on the magnitude, dynamics, and type of damage caused by infections. They also described later parabolic curves that explain the temporal evolution of host damage and symptomatic disease in the function of various host immune responses (13, 14, 29, 30). The microbiome, immunity, sex, temperature, environment, age, chance, history, inoculum, nutrition, and genetics (MISTEACHING) must be considered when applying this model to *P. salmonis* infections. Table 1 shows some of the questions raised by such an application and the possible investigations necessary to answer them (5, 6, 13, 14, 29, 30).

There are multiple possible causes for the dysbiotic microbiome in salmon with piscirickettsiosis: heavy use of antimicrobial/antiparasitic drugs, co-infections, stress, shortcomings in piscine nutrition, and aquatic environment eutrophication (5, 6, 15, 31–35). *P. salmonis* elicits a robust inflammatory response with significant tissue damage, but this response fails to clear the infection and is associated with high mortality (3, 5, 6, 16). While the salmon immune response remains mainly uncharacterized, preliminary evidence suggests granuloma formation in response to *P. salmonis* infection mediated by macrophages, B and T cells, and cytokines can produce inflammatory damage and necrosis in the kidney, liver, spleen, muscles, and skin.

The preferential use of females in salmon aquaculture to increase productivity and decrease aggressiveness may generate genetically homogenous individuals with increased susceptibility to developing piscirickettsiosis because of a lack of diversity in their immune response (5, 6, 16–18). Regarding temperature, wild fish may respond to infection behaviorally (13, 14, 36), but this option is unavailable for farmed salmon, and temperature may explain why *P. salmonis* infection and piscirickettsiosis are somewhat limited in the colder water areas of Southern Patagonia (19). Other environmental factors (stress from high concentrations of pens and fish in restricted areas resulting from overproduction based on economic goals, co-infections of fish with sea lice, algae, bacteria, and viruses, eutrophication, and dysbiosis of seawater secondary to heavy antimicrobial use) could provoke *P. salmonis* transition from a component of the microbiome to one associated with disease and, together with shortcomings in biosafety, facilitate the spread of *P. salmonis* secondary to clustering of pens and its high concentrations in seawater from farmed salmon with piscirickettsiosis in these pens (3–6, 10).

Although *P. salmonis* can be detected in the microbiomes of immature freshwater and adult fish, piscirickettsiosis tends to develop in juveniles recently transported from freshwater to seawater, indicative of the influence of age on the disease (3, 5, 6, 21–23). Age-mediated developmental changes in the immune response, microbiome, and metabolism could be responsible for these effects, as could the stress associated with increased biomass in pens (3, 5, 6, 16).

Chance may also play a role in the development of *P. salmonis* disease through the occurrence of mutations affecting antimicrobial resistance and *P. salmonis* virulence as well as through wind and currents that carry *P. salmonis* from salmon with piscirickettsiosis to uninfected fish (3, 6). In an environment containing many salmon cages, shedding of *P. salmonis* generates persistently high levels of potential inocula in the water, able to infect surrounding fish and produce disease despite *P. salmonis* being a tolerated component of the salmon microbiome and the salmon being healthy and immunocompetent (3, 5, 6, 10).

The natural history of salmon under aquaculture from egg to maturity and harvest is approximately 3 years. The origin of eggs and smolts and subsequent episodes of co-infections, stress, weight changes, and dysbiosis can all influence salmon infection by *P. salmonis* and the development of piscirickettsiosis (3, 5, 6, 21, 23). Co-infections may generate heterologous immunity that could protect from piscirickettsiosis as well as immunosuppression that would favor it, while weight increase could favor traumatic lesions in the skin secondary to encounters with the cage walls and other fish (5, 6, 16, 34).

The carnivorous diet of farmed salmon was initially based on fishmeal and fish oils but was shifted for economic reasons to contain more vegetable components (5, 6, 10, 15). This alteration could negatively affect the piscine immune response, metabolism, and integrity of skin and mucous membranes needed to avoid piscirickettsiosis following *P. salmonis* infection (5, 6, 15, 32). Accumulation of stools and uneaten food around salmon cages increases sources for eutrophication that may produce dysbiosis, anoxia, and algal toxicity which negatively affect salmon health and favor piscirickettsiosis (5, 6, 10). Nutritional immunity mechanisms may also play a role in blocking the development of piscirickettsiosis, as the genome of *P. salmonis* contains homologs of *fur,* a regulator of iron metabolism (3, 4, 16).

Finally, the genetic background of *P. salmonis* and its salmon host can influence the evolution of piscirickettsiosis. Even though no specific pathotypes of *P. salmonis* have yet been identified, the possibility of acquisition of new properties resulting from mutation and horizontal gene transfer could occur (4, 6, 10). While the genetic background of salmon under aquaculture is homogenous with a resultant decrease in variation in effective immune responses, their resistance to *P. salmonis* infections is polygenic, and their genetic manipulation is still complex (3, 16).

### Conclusions

**Fig 1:**
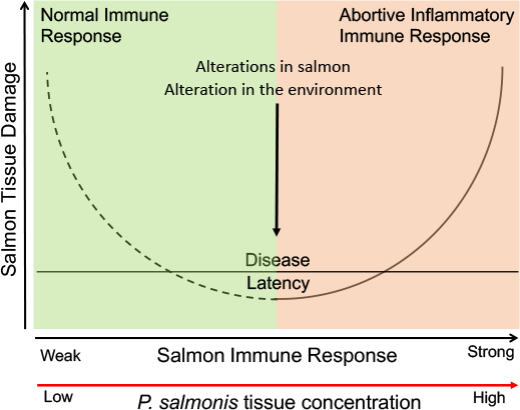
*Piscirickettsia salmonis* pathogenic potential. Alterations in the environment (eutrophication, algal blooms, fluctuations in temperature, hypoxia, biosafety lapses), in the salmon (microbiome, dysbiosis, immunity, sex, age, stress, co-infections, inoculum size, nutrition, genetics), chance and history, resulting in unrestrained and unbalanced bacterial multiplication and tissue damage (arrow). Experimental and clinical data indicate that tissue damage (right side of the parabola) is associated with a strong but incompletely effective immune response involving both antibody and cell-mediated elements. More knowledge is needed to ascertain whether the left side of the curve, represented by a broken line, will complete a parabola (29). While the usual presence of *P. salmonis* in the salmon microbiome might be beneficial to its eubiotic and balanced maintenance, whether its presence is associated with other potential damage or benefit awaits further knowledge. Figure based on Fig. 1 in reference 29.

Could the tissue damage in salmon caused by *P. salmonis* display a parabolic curve (Fig. 1)? While the numerous reports of such damage in salmon associated with cell-mediated immunity to *P. salmonis* is consistent with the right side of such a parabola (3, 4, 6, 29, 30), the relationship of the left side of such a parabola to *P. salmonis* infection remains undefined. This is because *P. salmonis* is a member of the salmon microbiome (3–6) and the type of damage/benefit, if any, produced at this stage of the infection is unknown (3–6, 29, 30). Could *P. salmonis* be a Class 6 DRF pathogen such as *Helicobacter pylori*? *H. pylori* infection is mostly asymptomatic and colonization probably benefits the human host by helping prevent acid esophagitis. Still, if *H. pylori* multiplies and the host mounts a strong immune response, gastritis and cancer are produced (13, 14, 30). In the case of *P. salmonis*, its residence in the salmon microbiome with apparently minimal tissue damage could be unbalanced by a solid but ineffective host immune response to its multiplication triggered by intrinsic and extrinsic factors in the salmon and/or the environment (5, 6, 10, 13, 14, 30).

Studies under the DRF to analyze and understand aspects of *P. salmonis* biology could help untangle the interactions in these processes, decrease the frequency of piscirickettsiosis, and lower the use of antimicrobials in aquaculture. They could determine and define the factors playing a role in the left side of the salmon damage/host response parabola (Fig. 1) (29, 30). They might help explain the failure of more than 30 vaccines to *P. salmonis* to protect against piscirickettsiosis by demonstrating immunotolerance to *P. salmonis* in salmon with these bacteria in their microbiome and why tetracycline and florfenicol fail to prevent or clear infection despite a widespread lack of any well-known mechanism of bacterial resistance (3, 4, 6, 13, 14, 29, 30). This latter failure could result from *P. salmonis* being an immunologically tolerated minor component of the microbiome (<0.01%), salmon defenses being overwhelmed by undetected frequent reinfections secondary to the high concentrations of shed bacteria in the ocean, novel antimicrobial resistance mechanisms (4, 6, 24), or combinations of all three. They might also clarify the factors involved in the differences in the frequency of piscirickettsiosis in Chile and Norway as well as the substantial contrast in antimicrobial use in the salmon industries of these two countries (6–8, 11, 12). Because of the failure of vaccines, antimicrobial treatments, and other measures to prevent and treat piscirickettsiosis despite this organism’s apparent lack of genetic recombination (4–6, 10, 16), such studies are important and urgently needed to prevent the appearance of strains with increased pan-antimicrobial resistance and virulence. They can be expected both to support the economic success and long-term sustainability of this critical industrial activity (6) and to have significant impacts on local and global piscine, human, and environmental health.
